# Privacy-Preserving Computation for Peer-to-Peer Energy Trading on a Public Blockchain

**DOI:** 10.3390/s23104640

**Published:** 2023-05-10

**Authors:** Dan Mitrea, Tudor Cioara, Ionut Anghel

**Affiliations:** Computer Science Department, Technical University of Cluj-Napoca, Memorandumului 28, 400114 Cluj-Napoca, Romania; dan.mitrea@cs.utcluj.ro (D.M.); ionut.anghel@cs.utcluj.ro (I.A.)

**Keywords:** secure multi-party computation, peer-to-peer energy trading, groups of prosumers, flexibility orders encoding, lower gas consumption, public blockchain

## Abstract

To ensure the success of energy transition and achieve the target of reducing the carbon footprint of energy systems, the management of energy systems needs to be decentralized. Public blockchains offer favorable features to support energy sector democratization and reinforce citizens’ trust, such as tamper-proof energy data registration and sharing, decentralization, transparency, and support for peer-to-peer (P2P) energy trading. However, in blockchain-based P2P energy markets, transactional data are public and accessible, which raises privacy concerns related to prosumers’ energy profiles while lacking scalability and featuring high transactional costs. In this paper, we employ secure multi-party computation (MPC) to assure privacy on a P2P energy flexibility market implementation in Ethereum by combining the prosumers’ flexibility orders data and storing it safely on the chain. We provide an encoding mechanism for orders on the energy market to obfuscate the amount of energy traded by creating groups of prosumers, by splitting the amount of energy from bids and offers, and by creating group-level orders. The solution wraps around the smart contracts-based implementation of an energy flexibility marketplace, assuring privacy features on all market operations such as order submission, matching bids and offers, and commitment in trading and settlement. The experimental results show that the proposed solution is effective in supporting P2P energy flexibility trading, reducing the number of transactions, and gas consumption with a limited computational time overhead.

## 1. Introduction

Digitization, decentralization, and engagement of small-scale energy prosumers in energy grid management are key features to ensure successful energy transition in Europe [1]. The participation of household prosumers in energy markets and demand response (DR) services is limited by both technological and human barriers [2]. With the adoption of the Internet of Things (IoT) energy meters, citizens have doubts about sharing their data due to privacy concerns, and in this aspect, consumers are supported, at least in Europe, by the General Data Protection Regulation (GDPR) [3]. At the same time, relying on a central platform for energy data management is not always reasonable, because it is exposed to attacks and data can be unintentionally leaked, which can result in serious security issues [4].

Blockchain technology is gaining popularity as a promising solution for eliminating the need for a central energy trading platform, allowing energy transactions to be recorded as immutable, shared transparently, and secured over a decentralized network. In such peer-to-peer (P2P) energy trading models, the prosumers can directly trade their energy surplus with other prosumers without centralized intermediaries to decrease costs and increase the local usage of renewables [5]. However, data privacy, low scalability, and transactional throughput are open challenges that need to be addressed for implementing blockchain-based decentralized energy markets.

For household prosumers, the mechanisms for privacy in P2P information sharing are relevant for their active participation in energy markets and effectively valorizing their flexibility to its full potential [6]. Without them, the attackers might obtain confidential information about the location of prosumers, personal information, energy consumption profiles, etc. [7]. Privacy solutions should consider the network and protocol levels for transactional data exchanges and the smart contracts that define, in lines of code, the terms of the P2P transactional energy agreement between prosumers [8]. Moreover, on a public blockchain, data are accessible to anyone who has access to the network and might violate privacy regulations [9]. In a P2P energy market implemented on top of the public blockchain, the prosumers submit energy bids and offers, which are stored and shared on the blockchain, matched in trades that represent commitments for delivery, and finally settled using the energy-monitored data [10,11,12]. All these operations are transparent for all participants, enabling the audit and validation using a standard procedure enforced by the smart contracts running on the blockchain. Due to their sensitive nature, the European Union has set clear rules and regulations regarding the privacy of prosumer data in the energy market [13,14]. However, they are not enough as modern Artificial Intelligence algorithms can infer energy consumption/production tendencies of household prosumers from energy trading data stored on the public blockchain and even find their true identities or behavioral patterns based on these values [15,16]. Moreover, an attack might further inject false information by generating false statistics from public data [17]. Mechanisms for privacy-preserving solutions are needed when deploying P2P energy markets using techniques such as encryption [18,19].

The P2P energy markets implemented on blockchain overlay experience problems related to scalability with an increasing number of prosumers and energy transactions [20]. A higher number of prosumers also increase the amount of transactional data to be stored and shared among blockchain network participants. The number of transactions to be processed and validated closer to real-time increase with the prosumers’ trading energy. Moreover, the encryption of transaction data usually leads to an increase in the size of the data leading to larger block sizes, thus more storage and more data sharing time are needed. However, many of the existing solutions address scalability by trading either security or decentralization of the blockchain or market implementation [21].

In this paper, we define a privacy-preserving wrapper for a blockchain-based P2P energy market [22] using the well-established secure multi-party computation (MPC) technique [23,24] to provide privacy and confidentiality guarantees for prosumers while trading their energy flexibility. The prosumers’ energy orders data represent a secret that is divided among different prosumers such that each one only stores one part of the secret, and no prosumer holds the entire energy order. As a result, the P2P energy market operations and associated computations can be performed on these shares without revealing the original energy order data. The technique allows for grouping prosumers and computing functions over the groups about the order data we are interested in, such as energy flexibility quantity and price. The groups of prosumers then participate in the P2P energy trading without revealing the group members’ identity, increasing the scalability of the energy flexibility market. As the energy transactions are carried out among groups, the number of transactions and blockchain gas consumption is reduced. Moreover, all the sensitive information is inferred or decoded from order shares and used for energy and financial settlement, so privacy is achieved in a decentralized energy market running on the blockchain. The advantage of the proposed solution is the intrinsic integration of secure MPC with the energy flexibility market described in [22], such that no changes are needed to the core market operation logic implemented in smart contracts. The encodings are performed off-chain while the transactional overhead is decreased by reducing the number of participants operating on the market. Moreover, compared with other cryptographic methods, such as homomorphic encryption [25], secure MPC uses less computing power as no encrypted numbers are used inside the smart contracts. The overhead regarding the secret sharing and function computation over them is limited, not affecting the market operation.

The rest of the paper is organized as follows: Section 2 presents the related work, Section 3 describes our approach for integrating MPC with P2P energy markets and enabling privacy and group-based flexibility trading, Section 4 and Section 5 show evaluation results and discuss benefits of our solution such as lower time and gas overhead, while Section 6 concludes the paper.

## 2. Related Work

We organized the state-of-the-art analysis into two main areas. The first direction focuses on solutions for ensuring privacy in P2P energy trading, while the second direction deals with solutions to enhance market scalability and transactional throughput.

The most encountered approach to ensure privacy in P2P energy trading is to encrypt the transactional data that is made public and shared on the blockchain. In [26], each user’s smart meter encrypts their data using changeable private keys, one for every other user it communicates with. The quantity of energy in each transaction is hashed to protect against statistical inference, and a method of authenticity verification without identifying it is proposed using a certificate of endorsement. Yahaya et al. proposed a two-layer, P2P energy, private trading system [27]. The first layer is an authentication layer, which prevents impersonation attacks. Two random numbers are used to generate public/private keys for buyers and sellers and to create and publish encrypted transactions. Using these parameters and a rule of regenerating new secret keys for each transaction, each entity can use them to verify the authenticity of the entity it trades energy with. If an authentication between two entities fails, the whole energy trading process is terminated. Electric Vehicles (EVs) are becoming an integral part of P2P energy trading and privacy is necessary. In [28] the authors tackled privacy issues in charging station-to-vehicle energy trading, as well as in vehicle-to-vehicle energy trading by encrypting information about both charging and discharging EVs, such as location, time, and amount of power transferred. To prevent Sybil attacks, a complex payment system is used which blocks attackers from linking EVs to their drivers. Radi et al. proposed a system where energy traders make bids to EVs owners, who can reserve their preferred charging station for a specific period [29]. Privacy is ensured by an anonymous payment system in which the payments are encrypted using blind signatures [30] and decrypted using the Schnorr protocol [31]. Similarly, in [32] a technique was proposed which utilizes higher degree polynomials that enable the execution of multiple Schnorr protocol instances at a close cost to a single instance. Umer et al. defined a coloring-based, fully decentralized communication algorithm, where an entity shares data only with its neighbors, significantly reducing required communications and thus, enhancing privacy and scalability [33]. In P2P energy trading the price and energy quantity must be shared, and by doing it only with each prosumer’s neighbors, greater privacy is ensured.

Zero-Knowledge Proofs (ZKPs) allow to prove that a statement is true without conveying any other information about that statement [19]. In [34] the authors leveraged this technique in a double auction market to increase privacy. The double auction technique consists of a closed bidding stage, and then an energy exchange stage. During the first stage, all users submit their bids on the blockchain, with the quantity hidden, together with a commitment, which is an encryption method. After matching, these commitments are verified based on ZKPs without revealing the bid quantities. Other authors use ring signatures to assure privacy in P2P energy trading [35]. Each transaction between parties must be signed by a minimum number of participants which can form up a public key. Then there is a minimum number of participants who can team up and redeem a signed transaction. Moreover, each entity broadcasts messages to all other participants, and each receiver tries to decrypt the message using its private key, thus the others do not know who exactly sent the message in the first place. To send a message, a proof-of-work algorithm must be executed to prevent spam. The linkable ring signature technique [36] allows to check if two signatures were generated for a message under the same public/private key pair and if yes, it proves dishonesty. In [37], this method is used to enable energy buyers to generate a one-time address and can only send one message with that address. With their public/private key, buyers encrypt a message only once, which should include a price list and energy quantities. If the same key pair is used for more than one message, the seller can check this by reconstructing the private key of the buyer and re-signing the message, but without revealing the buyer’s identity.

In [18,38], Function Hiding Inner Product Encryption technique [39] was used to encrypt order quantities and prices. First, order quantities are represented as vectors whose product tells us which vector is greater than the other. Then using this technique, encrypted quantities and prices can be ordered and matched, without revealing their values. In [40], Attribute-Based Encryption technique was used to perform encryption and allow users to decrypt energy data without the need of any third party. The secret keys are generated by a certificate authority and handed to all participants. Predicted values of production, together with price range and other data such as location of identity, are encrypted with this key. The buyer then searches for specific attributes and by using the above certificate, decrypts only the values which match their requirements, thus generating P2P energy trading in a secure manner. Finally, homomorphic encryption is used for P2P energy trading because it allows us to perform operations on encrypted data, the result is identical to performing these computations on raw data [41]. Computations can be performed without access to the secret key, which is necessary only if we want to see the result in raw format. In [42] the authors use this technique to ensure privacy of energy orders. Order prices are sorted by applying a sorting algorithm that uses homomorphic encryption and operations on encrypted data. After sorting the bid orders decreasingly and offers increasingly by price, members verify all transactions by checking the correctness using the public key.

The scalability in a P2P energy market implemented over blockchain overlays is challenging due to the tradeoffs that need to be made among decentralization, security, and scalability [20,43]. Moreover, decoupling between energy players and system-level markets might generate conflicts that need to be tackled. The scalability of the blockchain-based P2P energy market was analyzed in [44] using 37 household prosumers. Using Proof-of-Stake and varying the number of validators, the transaction throughput decreases significantly, while the mean latency is 10 s for the highest transactional throughput. Decentralized P2P energy markets can be developed using various blockchain technologies, such as a permissioned blockchain which uses another type of Byzantine fault tolerance. Abdella et al. unified three types of energy markets with a single payment system for a lower transaction amount [45]. By increasing the number of transactions, the latency also increases, the peak latency being met during market clearance and settlement phases. The scalability of structured and unstructured models for P2P energy trading were analyzed in [46]. The structured model organizes peers, and every peer has a list of its neighbors while for the unstructured model, the peers establish connections with others without any rules. Both models feature a linear increase in messages exchanges with the number of prosumers. Local Electricity Markets were virtually created in [47] by mixing complementary loads and renewable profiles and clustering the prosumers orders based on prices. Moreover, the bigger the community and renewable sources are, the higher the potential of cost saving is. There are trade-offs for EVs participation below a certain price threshold. Providing EVs with incentives based on their self-interest is a suitable technique for achieving balance between demand and response [48]. A double auction mechanism between EVs acting as buyers of energy, and service providers, who sell energy, preserves privacy, and maximizes social welfare, and is implemented through smart contracts. Nodes of the blockchain network act as energy brokers which manage local EVs. Scalability issues of the blockchain regarding P2P energy markets are addressed by implementing off-chain channels [49,50]. This model proposes four layers: a marketplace; off-chain channels for negotiation, main scalability influence; communication layer for entity grouping; and an electrical grid. Negotiation results between prosumers yield public transactions on the blockchain, so privacy is achieved by temporary unique identifiers which are encrypted. Scalability is increased due to lower blockchain transactions. Small-scale P2P energy trading can cause physical congestion during trading, and we need to avoid such cases [51]. A two-step process is implemented where, if a safety check fails, a second step kicks in and represents the network congestion management procedure. The method shows the same promising results in both 11-bus and IEEE 33-bus distribution systems.

The number of trading prosumers is the main factor in considering scalability, so reducing the number of players in a trading group can increase it and at the same time address the market entry barrier [52]. Segmentation time increases exponentially with the number of prosumers, but bilateral trading can be used for reducing the computational time in community-based markets. Adaptive segmentation is proposed as a P2P market clearing mechanism in [53]. The goal is to clear the market bids and offers with minimum data exchanged, also considering privacy and minimization of costs. Market segmentation uses similarities among players to group them using clustering to reduce the number of data shared outside the group. A similar model is proposed in [54] to enhance scalability. Prosumers are coupled in groups such that the profit value of groups is equal or has a slight difference. The profit value is computed based on the energy committed by prosumers as well as their location and energy type, renewable or not. The average response time and transaction time increase exponentially by the number of prosumers.

## 3. Materials and Methods

In this section, we start with a short outline of the P2P energy flexibility market used as support for the integration of the proposed privacy-preserving methods (Section 3.1), and then we focus on the novel contribution of this paper related to the grouping and encoding the prosumers’ energy orders using secure MPC and their integration with the smart contracts facilitating the P2P trading and market operation (Section 3.2 and Section 3.3).

### 3.1. P2P Energy Flexibility Market

In previous papers, we designed a local energy flexibility market for prosumers to trade their energy in a P2P manner [22]. We formalized two types of prosumers that can interact with the market. Energy consumers can be any energy asset with higher energy demand than production. Energy producers can be small households owning green energy devices and having a surplus of renewable energy.

The market is developed on top of a public blockchain (i.e., Ethereum) and smart contracts are defined to deal with the market session management. Upon initial registration with the market, the prosumers can submit offers for selling their surplus of energy or bids to buy the energy to cover their deficit. An energy forecasting service uses historical energy data and weather data to predict energy consumption/production of each prosumer for the next day. The information is used by prosumers to put orders for the next day of the market’s session. A module takes all these market orders to match the flexibility bids with flexibility offers and generates transactions that are inserted into the blockchain. The last step is the settlement of the transaction using the commitments registered in the trade placement phase and energy meters’ periodic measurements. These hourly measurements are compared with the committed values, and the energy and financial settlement of prosumers’ wallets via tokens distribution is performed.

**Figure 1:**
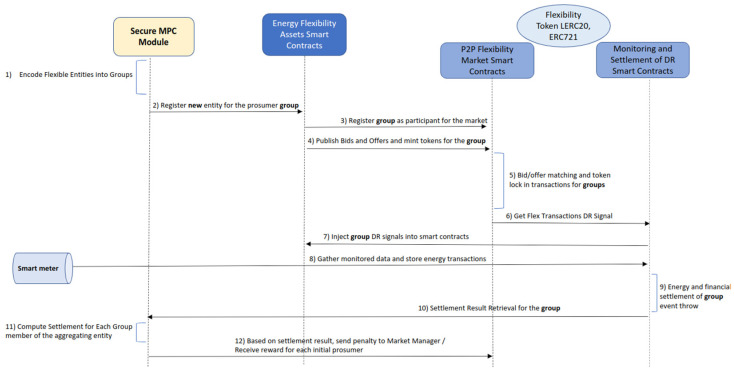
Energy flexibility market smart contracts and secure MPC integration.

All the above operations are made possible by smart contracts running on the Ethereum (see Figure 1). We have multiple types of smart contracts, each fulfilling a specific role. A market manager contract is responsible for registering prosumers in a market session and energy certificates are used for replacing the physical energy before the actual transfer. Furthermore, it launches the market sessions using a specific energy certificate previously registered. The market session smart contract enlists orders for each specific session and registers trades. Flexible entity smart contracts represent a flexible entity of a prosumer. They are the medium through which orders are placed for prosumers, and monitored values are stored on the chain. The smart contracts have rules for tracing prosumers energy consumption, generating energy flexibility orders, and saving commitments which can be later verified in the settlement phase using energy can be monitored by the smart meters.

We use a blockchain network to store the hourly energy data collected using smart meter devices installed in prosumers’ homes. Two types of tokens are used to digitize the energy as tokens ERC721 to make the assets traceable and ownable, and a Lockable ERC20 token for transferring fungible assets between prosumers.

In this paper, we go beyond our previous work on P2P energy trading by defining and integrating privacy-preserving mechanisms based on secure MPC. In previous work, all the prosumers’ energy orders are stored on the public blockchain to be verified and audited. However, this generates privacy concerns as the transactional data are transparent. Our solution leverages on secure MPC to group prosumers and encodes the energy orders on the market to make them private, and to allow to compare, match, and settle bids and offers in transactions. We obfuscate the amount of energy traded by the participants while all the needed operations, such as addition, multiplication with a constant, and comparison, are performed on encrypted values, using an implementation of Function-Hiding Inner Product Encryption [39]. As a result, the P2P energy market operations and associated computations are performed on these obfuscate data without revealing the original energy order data. The groups of prosumers participate in the P2P energy trading leveraging on smart contracts to submit orders in the market session and transaction settlement without revealing the group members’ identity, increasing the scalability of the energy flexibility market.

### 3.2. MPC-Based Flexibility Order Encoding

To encode the orders on the market, we use secure MPC. We want to hide the amount of energy that each market participant is trading by integrating the technique proposed [48]. Our goal is to obtain all the prosumers that want to submit energy flexibility orders on a market session and create random groups while mixing their submitted energy values. The prosumers are grouped considering the type of order either in groups of offers or groups of bids.

We represent a day ahead flexibility order using an array of 24 quantities of energy, one for each hour and a price for the entire order:

(1) $$P_{bid,i}=\left(E_{bid}\left(T\right),{Price}_{Gwei}\right),where\;E_{bid}\left(T\right)=\{e_{bid}\left(t\right):t\epsilon T=\left\{1,\ldots 24\right\}\}$$

(2) $$P_{offer,i}=\left(E_{offer}\left(T\right),{Price}_{Gwei}\right),where\;E_{offer}\left(T\right)=\{e_{offer}\left(t\right):t\epsilon T=\left\{1,\ldots 24\right\}\}$$

To encode orders on the energy market, we consider the total number of prosumers that want to place orders on the market session (N) and split into random groups of r members considering the order type (i.e., bids and offers). These groups can have any number of members r, if r is divisible with the total number of prosumers. In this way we assure that the groups created have an equal number of participants:

(3) $$Pros=\left\{P_{k}\right|k\in \left\{1\ldots N\right\},P_{bid,k}\vee P_{offer,k}⋈Market\;Session\}$$

(4) $$\left(G,⊣R\right)=\left\{\left(g_{j,type},R\right),\right|R=random\left(N\right),j=1\ldots k,type=bid\vee offer\}$$

where $\left(g_{j},r\right)$ is a group having r prosumers that submitted only bids or only offers on the market session. By joining all created groups, the initial set of prosumers on the market session should be obtained:

(5) $$Pros={⋃}_{j=1}^{k}\left(g_{j},R\right)$$

Inside a group, the bids and orders are spilt in random numbers R and the new values are distributed among the prosumers of the group. By splitting we mean that the sum of these random values is equal to the initial order value. We split the energy values considering each time slot t ϵ T as well as the order’s price:

(6) $$e_{bid}\left(t\right)=\{e_{bid}^{r}\left(t\right)|\sum_{n=1}^{r}e_{bid}^{r}\left(t\right)=e_{bid}\left(t\right)\}$$

(7) $$e_{offer}\left(t\right)=\{e_{offer}^{r}\left(t\right)|\sum_{n=1}^{r}e_{offer}^{r}\left(t\right)=e_{offer}\left(t\right)\}$$

(8) $${Price}_{Gwei}=\{{Price}_{Gwei}^{r}|\sum_{r=1}^{R}{Price}_{Gwei}^{r}={Price}_{Gwei}\}$$

Each member of the group splits its energy order, then it keeps only one of those random values and sends the rest to the other group members (i.e., one value is assigned to one member). As a result, each member of the group creates a new energy order by aggregating all the individual order values received form the other peer members of the group including its own random value:

(9) $$P_{orders,k}=\{e_{P_{j},r}|r\in 1\ldots R,P_{j}\in \left(g,⊣R\right),e_{P_{j},r}type=bid\vee offer\}$$

(10) $$P_{bid,k}=\sum_{r=1}^{R}e_{P_{j},r}|r\in 1\ldots R,P_{j}\in \left(G,⊣R\right),type=bid\}$$

(11) $$P_{offer,k}=\sum_{r=1}^{R}e_{P_{j},r}|r\in 1\ldots R,P_{j}\in \left(G,⊣R\right),type=offer\}$$

A similar approach on splitting, distributing, and aggregating among the group participants is used the price:

(12) $$P_{price,k}=\sum_{r}^{R}{Price}_{Gwei}^{r}(P_{j}),\in 1\ldots R,P_{j}\in \left(G,⊣R\right)$$

As a result, each prosumer has a new order comprised of an energy amount and price. The goal is to hide the energy values of each entity while operating on the market, so after executing the above mixing process, we have a single group operator G who acts on the market on behalf of the group. It can be viewed similarly as an energy aggregator who is assigned a random group, splits each participant’s order values, then operates on the energy market, and in the end, after the trading settlement phase is completed, distributes the rewards or penalties for each member according to their original promised values and actual delivery. The amount of energy of the group order is calculated as:

(13) $${\left(g_{j,type},R\right)}_{order}=<\sum P_{order,k},avg\left(P_{price,k}\right)>,\forall \;P_{k}\in \left(g_{j,type},R\right)$$

The energy tokens distribution needs to be performed per actual energy delivery to keep the market competitive and fair for participants. As the prosumers are clustered in groups and the initial energy orders are mixed by splitting, distribution, and aggregation, the new orders generated can be very different from the original ones. The prosumers with higher energy amounts have a higher contribution to the group, and the tokens during settlement need to be allocated accordingly. Thus, we privately associated the percentage from the group’s sum of each prosumer member. The process is formally described in Algorithm 1.
**Algorithm 1:** Formal description of the algorithm used for energy orders encoding.**Inputs:** Pros—the list of prosumers participating to the market session **Outputs:** ${\left(g_{j,order},R\right)}_{order}$—the prosumers groups bids and offers determined **Begin** **for each** prosumer *P* in Pros **do**  determine de sets of energy offers $P_{offer}$ and energy bids $P_{bid}$
**end for each**$R=random\left(N\right),order$**for each type** of order={offer,bid} **do**  **construct** groups of prosumers $\left(G_{order},⊣R\right)$  **for each** $\left(G_{order},⊣R\right)$ do    **split** $P_{order}$ in R parts $P_{offer,R}$    **distribute** $P_{order,R}$ to group $\left(G_{order},⊣R\right)$ prosumers    **destroy** initial offer $P_{order}$  **end for each**  **construct** ${\left(g_{j,order},R\right)}_{order}$ **us*ing*** $P_{order,R}$
**end for each****End**

To formalize the settlement process, we need to compute the percentage of each prosumer’s initial order from the order of the group and store it securely. We use the values monitored from each prosumer smart meter to compute the settlement of the group as its sum. Each prosumer’s individual monitored values are private. After knowing the settlement value of a group, for all 24 h of the day, we just need to compute each initial prosumer’s percentage from the value and use it as the payment of each initial prosumer. Finally, each group places the orders on the flexibility market using smart contracts.

### 3.3. Integration with P2P Trading Smart Contracts

To use the secure MPC implementation described above for P2P energy trading we modified the flexibility orders registration process to consider the creation of prosumer groups while leveraging on the functionality provided by the smart contracts in Figure 1. We capture the initial sets of bids and offers the prosumers are submitting to the market session and use the algorithm from Algorithm 2 to create groups based on the order type. Furthermore, for the created groups, new market participants are registered to the flexibility market and the aggregated orders are stored on the blockchain.
**Algorithm 2:** Encoding and placing flexibility orders with MPC.**Inputs:** P—the list of prosumers placing either bids OR offers $P_{bid}\;or\;P_{offer}$ **Outputs:** − **Begin** $r\leftarrow generate\;Random\;Number(1,P_{size}/2))$$number\;Of\;Groups=P_{size}/r$$List.shuffle\left(P\right)$$List<List<\gg Groups\leftarrow P.groupBy\left(i\to i\%number\;Of\;Groups\right).\;\;\;\;\;\;\;\;\;\;\;\;\;\;\;\;map\left(list\to list.stream()\right).collect(toList));$**for each** group∈Groups **do**  new Group SmartC←FlexibleEntitySC.deploy()  new Orders←COMPUTE_ORDER_VALUES(group)  $price\leftarrow \frac{SUM\left({group}_{prices}\right)}{{group}_{size}}$  **for each** member∈group **do**   LERC20SC.transfer(member, new Group SmartC)  **end for**  $MarketManagerSC.registerFlexibleEntity\left({new\;Group\;SmartC}_{address}\right)$  **for each** order∈new Orders **do**   $\left({new\;Group\;SmartC}_{address}\right).placeOrder(order,\;price)$
  **end for****end for****End**

We obtain the lists of prosumers who are placing either flexibility bids or offers in the current market session. We create separate groups for each category. A random number is generated for the group size between 1 and half of the number of prosumers (line 1). We divide the prosumers into equal groups by randomly shuffling the prosumers (lines 3–4). We are instantiating and deploying a new smart contract for the group to deal with the market interaction on behalf of the group such as placing orders (line 6). We use the P2P market contracts to register the new group as a Flexible Entity to be able to act on the market and place orders, because our energy market is private, and access needs to be granted by the Manager. The group order energy amount is computed by combining the order values of each prosumer member in the group and computing the median price for the flexibility using relations 10–12 (lines 7–8). This step is detailed in Algorithm 3. We transfer Lockable ERC20 tokens from each smart contract of the prosumers belonging to the group, to the smart contract owned by the group, such that it can submit the flexibility order in the market (9–14). Algorithm 3 shows the algorithm for generating new flexibility orders on behalf of the group (*COMPUTE_ORDER_VALUES*) after randomizing each energy amount value of prosumers.
**Algorithm 3:** Computing the flexibility order values for a group of prosumers.**Inputs:** G - group of prosumers, **Outputs:** $G_{flex-order}$ list of newly generated hourly flexibility orders **Begin**
$E_{flex}\leftarrow CREATE\_EMPTY\_LIST\_OF\_LISTS(G_{size})$**for each** p∈G **do**  ${orders}_{p}\leftarrow getOrders(p)$  $E_{flex}.add\left({orders}_{p}\right)$**end for**$G_{flex-order}\leftarrow CREATE\_EMPTY\_LIST()$**for each** $hour\in \left\{0,\ldots ,23\right\}$ **do**  ${orders}_{new}\leftarrow CREATE\_EMPTY\_LIST\_OF\_LISTS()$
  splitValues←CREATE_EMPTY_LIST_OF_LISTS()
  **for each** $listl:E_{estimated}$ **do**   currentValues←CREATE_EMPTY_LIST()   $value\leftarrow l_{hour}$
   **for each** $i\in \left\{0,\ldots ,r\right\}$ **do**
    **if** value≠0 **do**       ${num}_{random}\leftarrow GENERATE\_RANDOM(0,value)$       $currentValues.add({num}_{random})$       $value\leftarrow value-{num}_{random}$       **end if**
   **end for**   currentValues.add(value)
   $splitValues.add\left(currentValues\right)$
  **end for**  **for each** $i\in \left\{0\ldots r\right\}$ **do**    ${orders}_{new}.add\left(SUM\left(splitValues\left[i\right]\right)\right)$
  **end for**  $G_{flex-order}.add\left(SUM\left({orders}_{new}\right)\right)$**end for****return** $G_{flex-order}$
**End**

The algorithm takes as input the list of prosumers who form the group while the output value is the list of the newly generated group order values for each hour. We start by creating a list of the flexibility order values for each prosumer in the group (lines 2–5). We proceed by splitting the flexibility order values and then recombining them into new orders, whose sum represents the group’s orders (lines 8–9). For each hour of the day, we take each initial (i.e., before split and shuffle) flexibility order of all prosumers in the group and we split the energy values into *r* random parts such that their total remains equal the initial value (lines 13–19). We create a new data structure containing the list of randomly split values of each order value for the current hour, then we sum all values from this new data structure by obtaining the values from the lists at index 0. *r* to obtain the split order values of prosumers (lines 20–24). In the end, we sum these new split order values and obtain the group’s flexibility order values for each hour (line 26).

When the session ends, the matching and commitment phases begin using the flexibility orders submitted by the groups of prosumers created. We used the matching algorithm we defined in [22]. Two orders matched together result in an energy flexibility trade which is saved in the blockchain for each group of prosumers such that it can be verified later in the settlement phase. Each group should own and lock the flexibility they are trading in transactions using tokens.

Finally, prosumers’ energy monitored from the smart meters is used to perform market settlement. We define a smart contract method to register a monitored energy value hourly for each prosumer. We compare the monitored value to the promised one in the stored trades to make the tokens distribution accordingly. To ensure this market functionality we need to decode from the group flexibility orders the original percentual contribution of each prosumer (see Algorithm 4).
**Algorithm 4:** Computing the percentages of each order from the group total.**Inputs:** G – group of prosumers       $G.{order}_{values}$ – the order values of each prosumer in the group **Outputs:** percentages – the percentage contribution of each prosumer to the      group flexibility order **Begin**
percentages←CREATE_EMPTY_OF_LISTS()**for each** $i\in \left\{0\ldots r\right\}$**do**  sum←0
  **for each** hour∈{0…23} **do**    $sum\leftarrow sum+G.order_{value}\left[hour\right]$
  **end for**  currentPercentList←CREATE_EMPTY_OF_LISTS()
  **for each** hour∈{0…23} **do**   $percent\leftarrow \frac{G.order_{value}\left[hour\right]}{sum}*100$   $currentPercentList.add\left(percent\right)$
  **end for**  $percentages.add\left(currentPercentList\right)$**end for****return** percentages
**End**

This computes the order values for a group such that we know each participant’s energy quantities. We compute the sum of the prosumer’s flexibility orders and then the percentage of each one of them from the total (lines 2–10). We leverage on a smart contract method to throw events inside methods, events which we can query and see the values they exposed. During the settlement process, it is called each hour when energy monitoring is registered on the blockchain to determine the compensation payment.

## 4. Evaluation Results

In this section, we evaluate the above solution in the context of the decentralized P2P energy flexibility market aiming to assess if it can successfully support and assure privacy across all market operations from prosumers orders placement, token minting, trades matching, and settlement.

We considered the monitored infrastructure described in [22] to acquire energy data from several household prosumers: (i) three prosumers act as flexibility buyers who have more renewable energy and need supplementary demand to cover their surplus; and (ii) nine prosumers act as flexibility sellers, meaning that they can shift their flexible loads for limited intervals on request. For registering monitored energy values, we process smart meters’ data, aggregate them hourly, and insert them into the blockchain.

**Figure 2:**
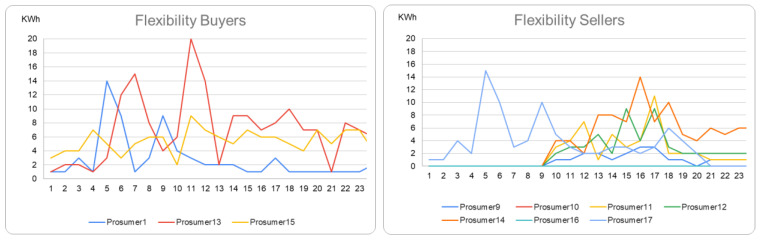
Energy profiles of the prosumers.

We set up a scenario in which the prosumers and their data are used to transact flexibility and transfer the tokens using the group structures defined using the secure MPC technique. Figure 2 shows the energy profiles of the buyers and sellers taking part in the scenario.

On the orders submitted by the prosumers we use the MPC-based encoding technique to aggregate them in groups of three and new group orders are placed into the market session such that sensitive information is not available regarding the identities of the prosumer’s participants. Table 1 shows the group orders created by shuffling all the prosumers and their original order flexibility amounts.

The initial energy order amount is split into three random values and shuffled between each group member. New order values are obtained for each prosumer and the prosumers join together in a group with the energy order value as the sum of the three initial prosumers (see Table 2).

**Figure 3:**
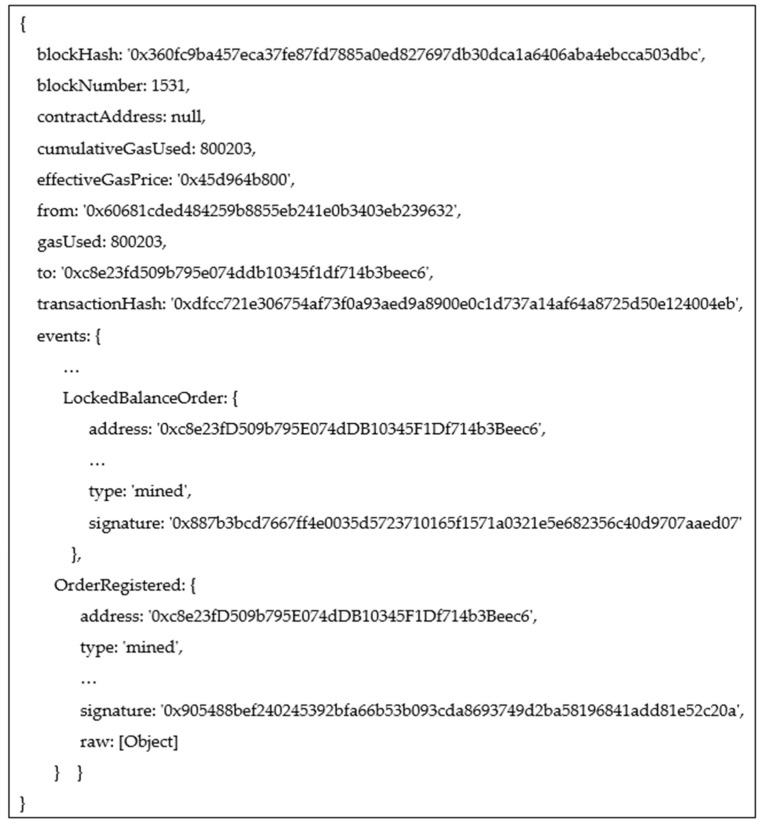
New order placement transaction and events thrown.

Before submitting the new orders to the market, each member of the group transfers the token amounts associated to the group smart contract. The tokens are locked in transactions until the settlement is performed. The orders are placed directly on the blockchain since the MPC-based obfuscation algorithm does not disclose information related to members of the groups created. It returns a set of buyer and seller prosumers who operate on the market. Figure 3 shows the blockchain transaction representing the group energy flexibility order and the events thrown.

**Figure 4:**
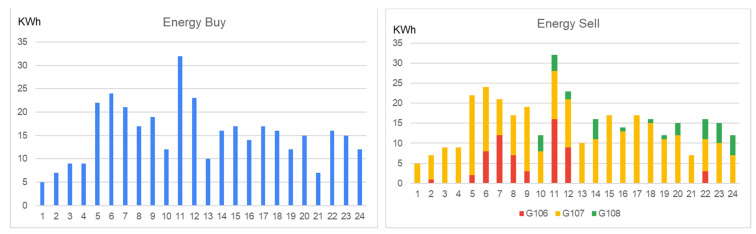
Matching the group energy flexibility and all orders in transactions.

After placing the energy flexibility orders for the next 24 h, the prosumers’ groups are matched in pairs of bids and offers. Figure 4 shows the result of the matching process for the defined scenario where the intersection of the bids and offers curves provides the reference price for energy flexibility trading.

**Figure 5:**
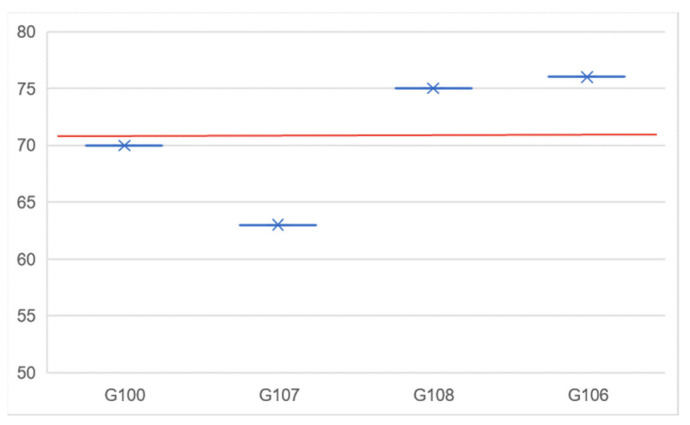
Group orders price and the trades reference price (with red).

These trades are inserted into the blockchain using one transaction to save gas by calling the method only once. The settlement price is determined as an average of the prices of the groups at the intersection of the bids and offers curves (Figure 5).

The blockchain smart contracts execute the commitment phase, saving on-chain, the energy each prosumer must deliver the next day. Each seller transfers their ERC721 tokens associated with the offer type orders to the buyers while the buyers transfer the locked ERC20 tokens associated with the quantity and price. In Table 3 we show the commitment values for groups of one buyer and three sellers, for the next day, as well as the token minted in the flexibility order placement phase.

**Figure 6:**
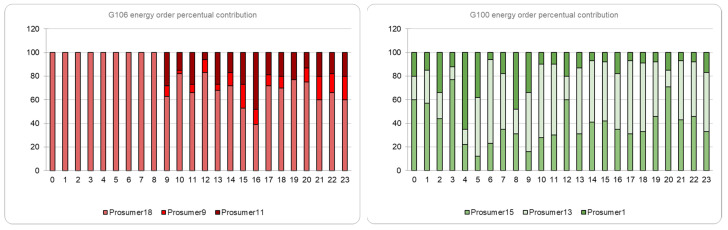
Group information and percentages contribution of prosumers to the total order amount.

For the settlement process, we register the hourly monitored values from energy meters and check if each group fulfills its trading commitment. The monitored energy values are compared with the committed ones in trades and the group’s payment amounts are computed. Afterward, the original percentage-based contribution of each prosumer of the group is decoded, and the payments are distributed accordingly (see Figure 6).

Table 4 shows the hourly monitored energy values for the group and their prosumers as well as the settlement payment of the group and distribution to member prosumers. As can be seen, the payment distribution from the group to prosumers is being performed considering the group-level settlement price and considers the penalties for prosumers that are not delivering as promised in the initial flexibility orders. In this case, we consider relevant only the deviations with more than 10% between the energy amount of the prosumer initial value and the actual monitored values.

## 5. Discussion

The proposed solution has clear benefits, not only for allowing us to obfuscate the energy profiles of prosumers on the public blockchain assuring the privacy of the confidential data but also for the energy market scalability, due to the reduced number of transactions on-chain. Our implementation adds two more wrapper layers, before and after the energy market, to fetch and process the data until the end of the market session. However, it generates groups acting on the market on behalf of a set of prosumers, thus resulting in a lower number of transactions stored on the chain.

**Figure 7:**
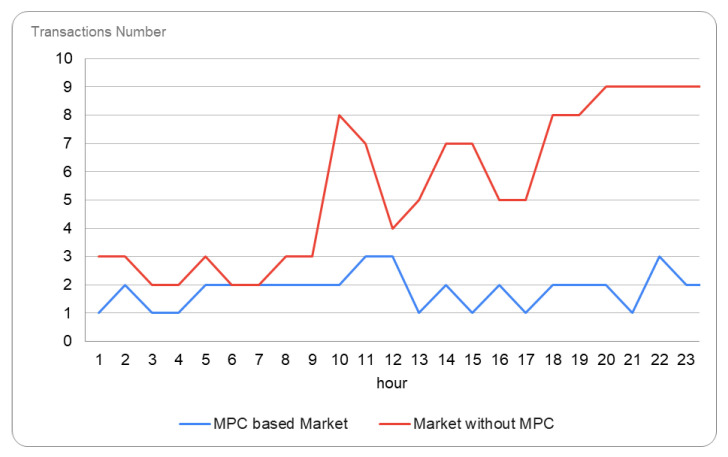
Number of P2P transactions over a day with and without MPC technique.

To compare our solution to an energy flexibility market with no MPC integration, we ran the same energy market but with all the prosumers acting directly on the market and going through all the market phases. Figure 7 shows the number of P2P transactions evolution in our scenario with and without the MPC-based technique. The number of transactions during a day in our scenario decreases from 132 transactions without MPC to 44 transactions with MPC, and on average from 6 to 2.

The number of market operations also decreases, the MPC-based implementation requiring three times fewer operations on-chain, which increases the performance overhead in regard to the blockchain network interaction. We compare these solutions both in terms of gas consumption on-chain, as well as time elapsed in each phase (see Table 5).

The market implementation without MPC consumes more gas in all the market phases. When considering the MPC solution, in the order placing phase, we also added the gas required for transferring the ERC20 tokens from the initial prosumers to the new, aggregating prosumer. However, we can see that the gas consumption is significantly lower in this solution compared with the classical solution, because we are performing the same operations, just for fewer orders. The same explanation goes for the trade registration phase too. We can see that gas consumption is much lower than in the classic market solution. The monitoring stage is the same, we have much fewer operations. The only difference in the monitoring phase between the MPC market and the classic solution, is that we perform the ERC transfer associated with rewards/payments after the monitoring phase in the smart contracts, but the gas consumption displayed above is computed from both stages and the result is much less than in the normal market.

Table 6 compares both energy markets implementation solutions in terms of processing time overhead in each phase. Both order placing phase and monitoring stage take three times less time to execute in the MPC market compared with the classic market. Although we have computational time overhead to group prosumers and compute their orders, these operations are executed relatively fast in any programming language.

The most time-consuming operation is the blockchain transaction registration, which takes up to 15 s to be executed and confirmed in a new block. Because all trades are registered in one transaction, the trade registration time is similar for the two solutions compared. So, no matter how many orders are submitted, we perform it in one transaction, though it takes the same amount of time to execute it. Regarding the other two phases, the processing time in the case of our proposed solution is less than a third of the time required in the market without MPC leading to scalability improvement.

## 6. Conclusions

In this paper, we proposed a secure MPC solution to provide privacy and confidentiality for prosumers during P2P energy trading while considering scalability issues. We provide privacy-preserving wrappers around an energy flexibility market core business logic enabling prosumers to create coalitions and trade flexibility as a group. Functions are defined and used to split prosumers’ order, encode the group flexibility order, and to trade on the market without revealing the identity of the group members. In this way, privacy is assured, no prosumer stores the entire order, and potentially sensitive information is inferred from order shares.

In the evaluation process, we obtained promising results. The proposed solution can be easily integrated with existing P2P markets with limited modification of the business logic implemented in smart contracts. Moreover, the matching of buy and sell orders in transactions and their energy and financial settlement was successful for both groups and prosumers. Our solution has a positive impact on energy market scalability by decreasing the number of transactions, gas consumption, and processing time in all market phases.

In the future, we plan to investigate the practicality of implementing our solution for multi-energy markets designed for integrated energy systems by exploring the possibility of trading not only electrical energy but also other forms of energy carriers such as heat or gas. Furthermore, we will consider other criteria for P2P energy trading, such as physical network limitations and the effects of randomly grouping prosumers on the level of flexibility.

## Figures and Tables

**Table 1:** Group order total flexibility amounts for 24 h.

Prosumers ID	Order Amount (kWh)	Group ID/ Order Type	Order Amount (kWh)
Prosumer1	68	G100/ Flexibility Buy	367
Prosumer15	169		
Prosumer13	130		
Prosumer9	21	G106/ Flexibility Sell	268
Prosumer18	199		
Prosumer11	48		
Prosumer17	85	G107/ Flexibility Sell	282
Prosumer12	52		
Prosumer19	145		
Prosumer14	21	G108/ Flexibility Sell	96
Prosumer10	44		
Prosumer16	31		

**Table 2:** Prosumers orders splitting in group G100 for hour 4:00.

	Prosumer 1	Prosumer 15	Prosumer 13	New Energy Orders
Initial Order (kWh)	14	3	5	G100 = 22
Order Split (kWh prosumer destination)	7 (Prosumer1)	0 (Prosumer1)	1 (Prosumer1)	Prosumer1 = 8
	3 (Prosumer15)	2 Prosumer15)	1 (Prosumer15)	Prosumer15 = 6
	4 (Prosumer13)	1 (Prosumer13)	3 (Prosumer13)	Prosumer13 = 8

**Table 3:** Commitment values for matched groups.

Hour	Flexibility Amount	L-ERC 20 Tokens	Group Buyer ID	Group Seller ID	Flexibility Amount	Group Seller ID	Flexibility Amount	Group Seller ID	Flexibility Amount
1	5	350	G100	G106	0	G107	5	G108	0
2	7	490			1		6		0
3	9	630			0		9		0
4	9	630			0		9		0
5	22	1540			2		20		0
6	24	1680			8		16		0
7	21	1470			12		9		0
8	17	1190			7		10		0
9	19	1330			3		16		0
10	12	840			0		8		4
11	32	2240			16		12		4
12	23	1610			9		12		2
13	10	700			0		10		0
14	16	1120			0		11		5
15	17	1190			0		17		0
16	14	980			0		13		1
17	17	1190			0		17		0
18	16	1120			0		15		1
19	12	840			0		11		1
20	15	1050			0		12		3
21	7	490			0		7		0
22	16	1120			3		8		5
23	15	1050			0		10		5
24	12	840			0		7		5

**Table 4:** Monitoring and settlement values for G100 and distribution to prosumer members.

	Monitored Values				Penalty				Settlement Payment			
Hour	Prosumer1	Prosumer15	Prosumer13	G100	Prosumer1	Prosumer15	Prosumer13	Prosumer100	Prosumer1	Prosumer15	Prosumer13	G100
1	1	3	1	5	0	0	0	0	71	213	71	355
2	1	3	2	6	0	71	0	71	71	213	142	426
3	3	4	3	10	0	0	71	71	213	284	71	568
4	1	9	3	13	0	142	142	284	71	355	−71	355
5	14	10	3	27	0	355	0	355	994	0	213	1207
6	7	8	11	26	142	355	0	497	497	−142	852	1207
7	2	5	16	23	71	0	0	71	0	355	1065	1420
8	4	2	11	17	71	284	213	568	142	142	355	639
9	9	14	5	28	0	568	71	639	639	−142	213	710
10	3	0	6	9	71	142	0	213	213	0	426	639
11	3	0	19	22	0	639	0	639	213	0	1420	1633
12	5	0	9	14	213	497	355	1065	−71	0	639	568
13	2	0	2	4	0	426	0	426	142	0	142	284
14	2	0	9	11	0	355	0	355	142	0	639	781
15	1	4	9	14	0	213	0	213	71	284	639	994
16	1	4	7	12	142	0	0	142	−71	426	497	852
17	3	0	8	11	0	426	0	426	213	0	568	781
18	3	2	10	15	142	213	0	355	−71	142	710	781
19	0	4	8	12	71	0	71	142	0	284	426	710
20	0	3	7	10	71	274	0	345	0	223	497	720
21	0	0	0	0	71	355	71	497	0	0	0	0
22	0	0	8	8	71	497	0	568	0	0	568	568
23	0	0	7	7	0	497	0	497	71	0	497	568
24	0	0	7	7	142	142	71	355	0	142	355	497

**Table 5:** Gas consumption comparison.

Gas Consumption—Gwei		
Market Phases	Market without MPC	MPC-Based Market
Order Placing	243,919,476	85,572,688
Trade Registration	123,665,425	44,042,230
Monitoring Stage	27,130,506	6,607,817

**Table 6:** Processing time comparison.

Time Elapsed—Millis		
Market Phases	Market without MPC	MPC-Based Market
Order Placing	1,440,256	541,109
Trade Registration	5967	4644
Monitoring Stage	1,497,439	538,901

## Data Availability

Not applicable.
